# Dietary Methionine Hydroxy Analog Regulates Hepatic Lipid Metabolism via SIRT1/AMPK Signaling Pathways in Largemouth Bass *Micropterus salmodies*

**DOI:** 10.3390/biology14030227

**Published:** 2025-02-21

**Authors:** Ju Zhao, Zhongjie Yang, Haifeng Liu, Chao Yang, Yujun Chen, Quanquan Cao, Jun Jiang

**Affiliations:** 1 College of Animal Science and Technology, Sichuan Agricultural University, Chengdu 611130, China; 2024102020@stu.sicau.edu.cn (J.Z.); 2024302171@stu.sicau.edu.cn (Z.Y.); liuhf@sicau.edu.cn (H.L.); 2018302040@stu.sicau.edu.cn (C.Y.); 2024202073@stu.sicau.edu.cn (Y.C.); 2 Fish Nutrition and Safety Production University Key Laboratory of Sichuan Province, Sichuan Agricultural University, Ya’an 625014, China

**Keywords:** methionine hydroxy analog, lipid metabolism, liver, largemouth bass

## Abstract

This experiment was arranged to explore the impacts of dietary MHA on liver lipid metabolism in largemouth bass. A total of 480 fish (14.49 ± 0.13 g) were randomly allocated into four groups, each with three replicates. They were then given four different diets containing graded levels of MHA (0.0, 3.0, 6.0, and 9.0 g/kg) for 84 days. The results showed that dietary MHA increased hepatic lipid vacuoles and lipid content (*p* < 0.05). Dietary supplementation with MHA 9.0 g/kg diets increased the activities of acetyl-coA carboxylase (ACC), fatty acid synthase (FAS), and stearoyl-coA desaturase 1 (SCD-1). Dietary MHA up-regulated the mRNA expressions of liver lipid synthesis (ACC, FAS, SCD-1 and SREBP-1c) (*p* < 0.05). Furthermore, compared with the 0.0 g/kg diet group, the group supplemented with 9.0 g/kg MHA in the diet exhibited a significant decrease in the activities of liver lipid-oxidation-related enzymes (acetyl-CoA carboxylase (ACC), fatty acid synthase (FAS), and stearoyl-CoA desaturase 1 (SCD-1), as well as HSL and CPT1) and the gene expressions of ATGL, HSLa, HSLb, CPT1a, and PPARα (*p* < 0.05). Additionally, the mRNA expressions and protein levels of SIRT1 and AMPK in the 9.0 g/kg MHA-supplemented group were significantly lower than those in the 0.0 g/kg diet group (*p* < 0.05). Overall, the present results suggested that dietary MHA could increase lipid accumulation through regulating SIRT1/AMPK signaling pathways in the livers of largemouth bass.

## Simple Summary

MHA serves as a functional and economical Met source. Prior studies show that dietary Met affects fish liver lipid metabolism, but MHA’s impact on largemouth bass liver lipid metabolism remains unstudied. Therefore, we investigated whether dietary MHA increased hepatic lipid vacuoles and content. A total of 9.0 g/kg MHA in a diet boosted ACC, FAS, and SCD-1 activities and lipid synthesis gene expressions. It reduced lipid oxidation enzyme activities and related gene expressions, and decreased SIRT1 and AMPK levels. We concluded that MHA promotes lipid accumulation via SIRT1/AMPK in largemouth bass livers.

## 1. Introduction

2-Hydroxy-4-(methylthio)butanoic acid, a methionine hydroxy analog (MHA), has been utilized more and more frequently in aquaculture feeds as a functional and cost-effective source of methionine (Met). Previous research conducted in our laboratory has demonstrated that dietary supplementation with MHA can potentially confer benefits in terms of growth, intestinal antioxidant capacity, and the intestinal microbiota of largemouth bass (*Micropterus salmoides*) [1]. The liver is an important organ involved in digestion, metabolism, and detoxification in fish [2,3,4]. In fish, excessive lipid accumulation in the liver can lead to hepatic damage [5]. A study has shown that a partial restriction of dietary methionine reduces lipid deposition in the livers of mice [6]. Conversely, dietary supplementation with Met has been shown to promote liver lipid accumulation in tiger puffer (*Takifugu rubripes*) and cobia (*Rachycentron canadum*) [5,7]. Nevertheless, the understanding of whether MHA can regulate lipid metabolism in fish remains limited.

Lipid accumulation in the liver is mainly related to lipid synthesis and degradation [8,9]. Sterol regulatory element-binding protein-1c (SREBP-1c) is a membrane-bound transcription factor, which regulates the expression of target genes involved in lipid anabolism, including acetyl-coA carboxylase (ACC), fatty acid synthase (FAS), and stearoyl-coA desaturase 1 (SCD-1) [7,10]. Adipose triglyceride lipase (ATGL) and hormone-sensitive lipase (HSL) play a decisive role in lipid mobilization and are the key enzymes of triglyceride hydrolysis [11]. Peroxisome proliferator activated receptor α (PPARα) is a critical transcriptional regulator, which stimulates the genes involved in mitochondrial fatty acid oxidation, such as aconitase, and carnitine palmitoyl acyl-coA transferase-1 (CPT-1) [12]. Aissa et al. (2014) reported that dietary Met supplementation improved the mRNA expression of SREBP-1 and decreased the mRNA expression of PPARα, which resulted in abnormalities in the lipid metabolism in mice [13]. A study on rainbow trout (*Oncorhynchus mykiss*) demonstrated that a partial Met restriction down-regulated the mRNA expression of FAS and SREBP-1, while up-regulating the PPARα expression [14]. Similarly, in cobia, a dietary Met deficiency suppressed the expression of genes associated with hepatic de novo lipogenesis (FAS, SCD-1, ACC1, SREBP-1) and up-regulated the PPARα expression [7]. The Met up-regulated the mRNA expression of ATGL in both yellow catfish (*Pelteobagrus fulvidraco*) and the HepG2 cells line [15]. In juvenile tiger puffers, high dietary methionine levels significantly enhanced the hepatic mRNA expression of HSL [5]. Furthermore, a Met supplementation markedly increased the hepatic mRNA expression of PPARα in blunt snout bream (*Megalobrama amblycephala*) [16]. These results suggested that Met has a regulatory effect on lipid metabolism. Nevertheless, so far, there is no information available regarding the effect of dietary MHA on lipid metabolism in fish.

Silent information regulator T1 (SIRT1) is a member of the NAD-dependent family of protein deacetylases and plays a crucial role in regulating hepatocyte lipid metabolism by activating AMP-activated protein kinase (AMPK) [17,18,19]. It has been reported that a partial restriction of dietary Met up-regulated the expression of SIRT1 in the kidneys of mice [20,21]. Related research also reported that when mice were fed with Met dissolved in water, the expression of SIRT1 in the aortic endothelium was repressed [20]. In rainbow trout, a dietary Met deficiency inhibits lipogenesis, probably due to the activation of AMPK [14,22,23]. Met deficiency significantly increases AMPK phosphorylation in the muscle cells of turbot (*Scophthalmus maximus*). However, the knowledge about the effect of MHA on the SIRT1/AMPK signaling pathway remains scarce. Whether the MHA can modulate lipid metabolism through the SIRT1/AMPK signaling pathway in fish remains to be investigated.

Largemouth bass is a freshwater carnivorous fish that is widely farmed in China, with the production of 619,519 tons in 2020 [24]. Our previous study demonstrated that dietary MHA, as a source of Met, promoted growth and modified intestinal microbiota diversity and composition [1]. Based on these findings, we hypothesized that MHA could also influence hepatic lipid metabolism in fish. To test this hypothesis, this study investigated the impact of MHA on lipid metabolism in the liver of largemouth bass. These results may provide a theoretical foundation for understanding the potential regulatory mechanisms by which MHA affects hepatic lipid metabolism.

## 2. Materials and Methods

### 2.1. Experimental Diets, Feeding Trial and Sampling

All experimental procedures used were conducted with the approval of the Animal Care Advisory Committee of Sichuan Agricultural University (Permit NO. DKY-2018202027). This experiment followed the same feeding trial setup and feed formulation as our previous study [1] (Table 1). MHA was added to the experimental diets at levels of 0.0 (control), 3.0, 6.0, and 9.0 g/kg. The Met content measured in the four experimental diets was 8.3, 11.0, 13.6 and 16.3 g/kg of dry diet (Table 2). A total of 480 juvenile largemouth bass of similar sizes (14.49 ± 0.13 g) were randomly assigned to 12 concrete tanks (2 m × 1 m × 1.05 m), with each replicate containing 40 fish. Throughout the feeding trial, the fish were hand-fed daily until they showed signs of apparent satiation at 08:00 and 18:00, following the natural photoperiod. Forty minutes after each feeding session, any uneaten feed was collected, dried, and weighed. These data were then used to calculate the feed intake. The feeding period was 12 weeks. At the end of the growth experiment, fish were starved for 24 h. Afterward, fish were anesthetized with benzocaine solution (50 mg/L). Livers from nine largemouth bass per tank were sampled and quickly frozen in liquid nitrogen, then transferred to −80 °C for the subsequent biochemical analysis, lipid extraction, real-time quantitative PCR, and Western blot assays. Livers from three fish per tank were preserved in 4% paraformaldehyde for histological examination.

### 2.2. Biochemical Analysis

The proximate composition of the diets was analyzed following the previously established methods [25]. Hepatic lipids were extracted using a 2:1 chloroform-methanol method [26]. Liver samples were homogenized in ice-cold physiological saline solution (10 volumes, *w*/*v*) and then centrifuged at 3000 rpm and 4 °C for 20 min. The supernatant was collected for enzyme activity analysis. Protein content was determined by the Bradford method [27]. The activities of ACC (H232-1-1), FAS (H231-1-1), SCD-1 (H648-1-1), HSL (H238-1-1), and CPT-1 (H230-1-1) were measured by an enzyme-linked immunosorbent assay (ELISA) using commercial detection kits (Nanjing Jiancheng Bioengineering Institute, Nanjing, China).

### 2.3. Hepatic Histological Analysis

The processes of dehydration, dyeing, and image collection were performed [28]. After the fixation, the livers were dehydrated by the standard procedures, embedded in paraffin, and cut to 5 μm sections. A total of 9 fish per treatment (3 fish per tank) were stained with Hematoxylin eosin (H&E), to evaluate the liver morphology, and observed in light microscopy. The areas of lipid vacuoles in the H&E sections were evaluated using Image J software (version 1.42, National Institutes of Health, USA), as described by [29].

### 2.4. Real-Time Quantitative PCR

The total RNA from liver tissue was isolated using the TRIzol reagent (TaKaRa, Liaoning, Dalian, China) and reverse-transcribed into cDNA using the PrimeScript^®^ RT reagent Kit (TaKaRa). The Nano Drop^®^ 2000 spectrophotometer (Shanghai Musen Biotechnology Co., Ltd., Shanghai, China) and agarose gel (1.5%) electrophoresis were used to test the RNA quantity and quality, respectively. RT-qPCR was conducted using the SYBR green method and the CFX96 Real-Time PCR Detection System (Bio-Rad, Hercules, CA, USA). The gene-specific primers and optimal annealing temperatures used in this study are shown in Table 3. The β-actin and 18S rRNA were used as the internal control. The mRNA abundances of target genes were calculated according to the 2^−ΔΔCT^ method [30].

### 2.5. Protein Extraction and Western Blot Analysis

The liver tissues were lysed with RIPA buffer (Beyotime, Shanghai, China). The protein concentration was determined by a BCA protein assay kit (Beyotime, Shanghai, China). Protein extracts (20 μg) were separated by 12% SDS-PAGE electrophoresis and then transferred onto polyvinyl difluoride (PVDF) membrane. After being blocked with 5% bovine serum albumin (BSA) in PBS, the PVDF membranes were dipped in the diluted primary antibody overnight at 4 °C. The anti-SIRT1 (1:1000, A11267, anti-rabbit), anti-AMPK (1:1000, A1229, anti-rabbit) were purchased from ABclonal Biotechnology Co., Ltd. (Wuhan, Hubei, China) The P-AMPK (1:1000; Thr172, AF3423, anti-rabbit) was purchased from Affinity Biosciences. The β-actin (1:1000; R23613, anti-rabbit, Zen Biotechnology, Chengdu, Sichuan, China) was used as the control protein. After primary antibody incubation, the membranes were washed by TBS/T and then incubated with second antibodies (1:2000; Zen Biotechnology, Chengdu, Sichuan, China). Finally, the protein was visualized by enhanced chemi-luminescence, then quantified by the Gel-Pro Analyzer (Media Cybernetics, Bethesda, MD, USA).

### 2.6. Statistical Analysis

The normality and homogeneity of the data were verified using the D’Agostino-Pearson and Bartlett tests. Data were analyzed using one-way analysis of variance (ANOVA), using SPSS 25.0 statistical software (IBM, Michigan Avenue, Chicago, IL, USA). The difference among treatments was examined using the Duncan’s multiple-range test. Orthogonal polynomial contrasts were used to determine the linear and quadratic effects of dietary MHA inclusion levels. The results were presented as the mean and the standard error of the mean (SEM). Statistical significance was set as *p* < 0.05.

## 3. Results

### 3.1. Hepatic Steatosis and Lipid Content

**Figure 1:**
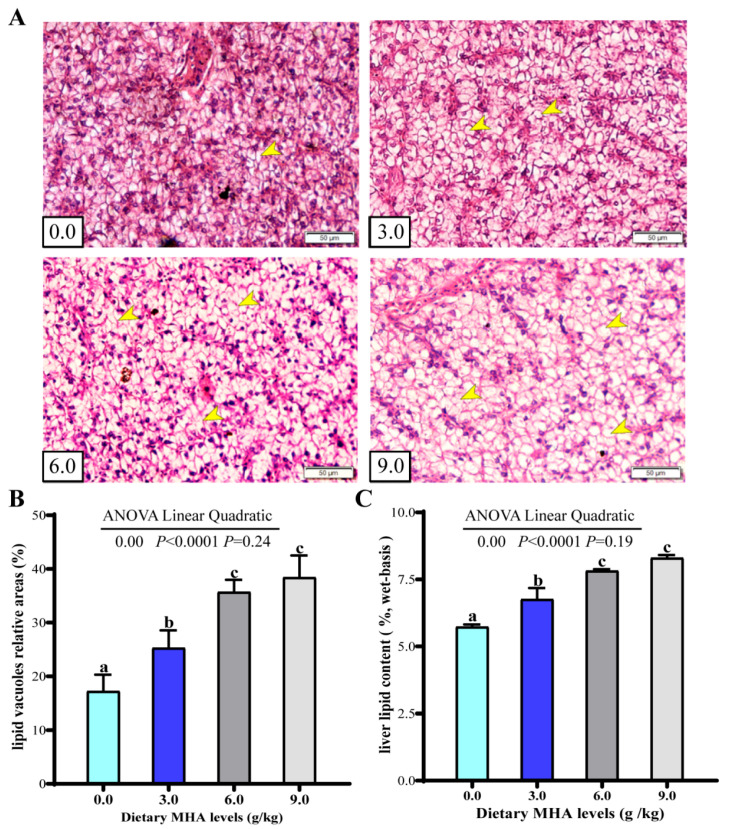
Effects of dietary MHA on lipid accumulation in liver largemouth bass fed diets with graded levels of MHA (g/kg) for 84 days. (**A**) Representative photomicrographs of histological alterations in the liver, stained with H&E staining at 200× magnification. The yellow arrow indicated the lipid vacuoles; (**B**) Relative areas for lipid vacuoles in H&E staining section (*n* = 9); (**C**) Lipid content of the liver (*n* = 9); Different letters denote significant differences (*p* < 0.05).

The hepatic microanatomy is illustrated in Figure 1A. Compared with the control group and the group fed with 3.0 g MHA kg/diet, the groups fed with 6.0 and 9.0 g MHA/kg diets showed a dramatic increase in the size and number of lipid droplets, characterized by hepatocytes filled with lipid vacuoles and a modified nucleus position. The orthogonal polynomial contrasts indicated that the increasing MHA levels linearly increased the relative areas of hepatic lipid vacuoles and hepatic lipid content (Figure 1B,C, *p* < 0.05).

### 3.2. Lipid Synthesis-Related Parameters in Liver

**Figure 2:**
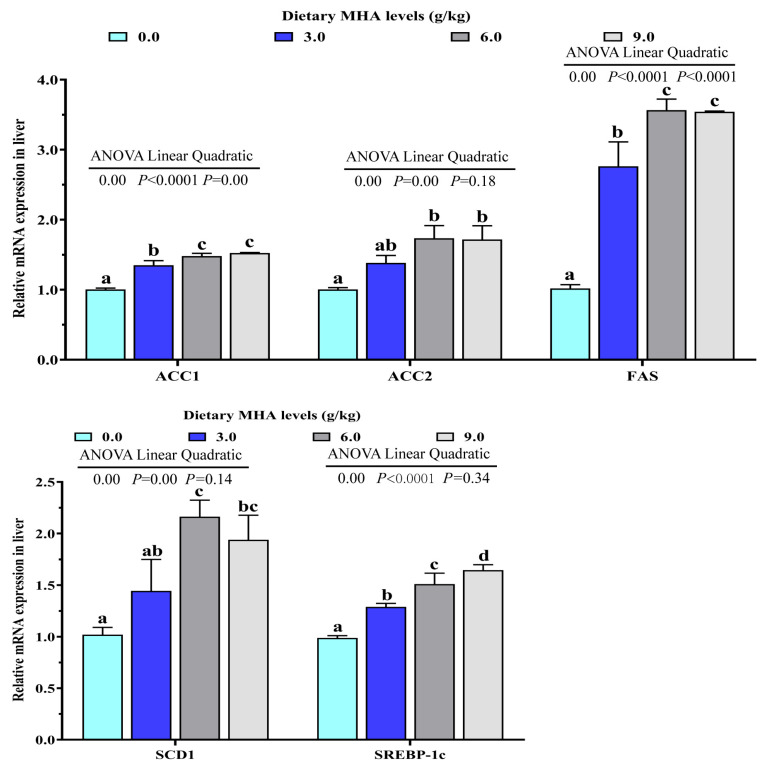
Effects of dietary MHA on lipid synthesis related gene expressions of ACC1, ACC2, FAS, SCD1 and SREBP-1c in livers of largemouth bass fed diets containing graded levels of MHA (g/kg) for 84 days. Means within columns with different superscript letters show significant differences at *p* < 0.05.

As shown in Table 4, the activities of ACC and SCD-1 increased linearly with the increasing dietary MHA levels (*p* < 0.05). The FAS activity increased significantly in both the linear and the quadratic manner (*p* < 0.05). The expressions of genes involved in hepatic lipid synthesis are presented in Figure 2. There was a linear and quadratic effect of dietary MHA level on mRNA expressions of ACC1 and FAS (*p* < 0.05). As dietary MHA levels increased, the mRNA expressions of ACC2, SCD1, and SREBP-1c increased linearly (*p* < 0.05).

### 3.3. Lipid Oxidation-Related Parameters in Liver

**Figure 3:**
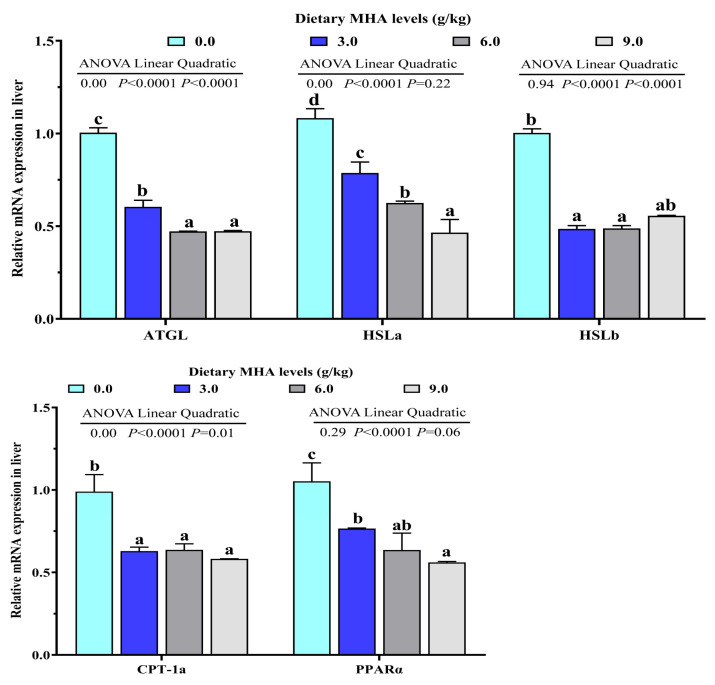
Effects of dietary MHA on lipid oxidation-related gene expressions ATGL, HSLa, HSLb, CPT-1a and PPARα in livers of largemouth bass fed diets containing graded levels of MHA (g/kg) for 84 days. Different letters denote significant differences at *p* < 0.05 (*n* = 9).

With an increasing dietary MHA level, the HSL and CPT-1 activities decreased linearly (*p* < 0.05). The mRNA expressions of ATGL, CPT-1a and HSLb were linearly and quadratically decreased with increasing dietary MHA levels (*p* < 0.05). The mRNA expressions of HSLa and PPARα decreased linearly (*p* < 0.05, Figure 3).

### 3.4. Dietary MHA Down-Regulated Hepatic SIRT1/AMPK Signaling Pathway

**Figure 4:**
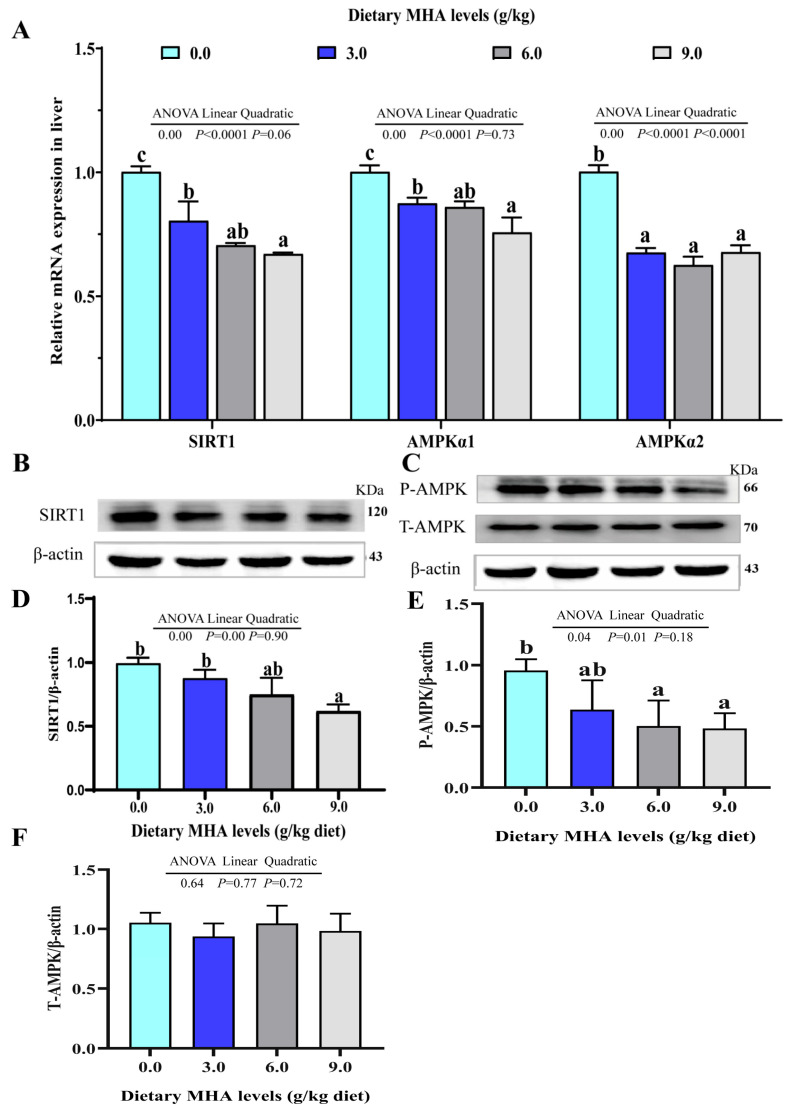
Effect of dietary MHA on the SIRT1/AMPK signaling pathway in the livers of largemouth bass fed diets with graded levels of MHA (g/kg) for 84 days. (**A**) The mRNA expressions of SIRT1, AMPKα1, and AMPKα2; (**B**–**D**) Protein abundances of SIRT1, β-actin, and ratio of SIRT1 and β-actin; (**C**,**E**,**F**) Protein abundances of P-AMPK, T-AMPK, and β-actin, and ratio of P-AMPK and T-AMPK. Different letters denote significant differences at *p* < 0.05 (*n* = 9).

As presented in Figure 4A, the orthogonal polynomial contrasts showed that the increasing MHA levels linearly and quadratically decreased the mRNA expression of liver AMPKα2 (*p* < 0.05). The mRNA expressions of SIRT1 and AMPKα1 decreased linearly (*p* < 0.05). The ratio of SIRT1/β-actin, P-AMPK/β-actin, and T-AMPK/β-actin linearly decreased with increasing dietary MHA levels (Figure 4B–F, *p* < 0.05).

## 4. Discussion

The digestive and metabolic functions of fish are largely dependent on the structural integrity of the liver, which is responsible for fat synthesis and storage [31,32]. Excessive lipid accumulation in the liver can be detrimental to liver health and, consequently, affect the growth performance of fish [5,33,34]. In the current study, as the dietary levels of MHA increased, the lipid content in the livers of fish also increased. In this research, this was further corroborated by a histological examination of the liver H&E-stained sections. Higher dietary MHA levels led to an increase in the number of lipid vacuoles within hepatocytes. Collectively, these results indicated that dietary MHA could promote lipid accumulation in the liver. Previous studies on tiger puffers and cobia demonstrated that dietary Met increased hepatic lipid deposition [5,7]. Similarly, Craig et al. (2013) reported that a dietary Met restriction decreased lipid content in liver of rainbow trout [14]. A study conducted on mice also found that a dietary Met restriction limited lipid deposition in liver [6]. Nevertheless, the detailed reasons require further investigation.

Lipid accumulation in the liver is primarily influenced by lipid synthesis and degradation [8,9]. ACC and FAS are the key rate-limiting enzymes in long-chain fatty acid biosynthesis [35,36]. SCD1 is required for biosynthesis of the long-chain monounsaturated fatty acids [37]. On the other hand, HSL and CPT1 are involved in lipolysis and fatty acid β-oxidation, respectively [15,38]. In the present experiment, the activities of ACC, FAS, and SCD1 significantly increased linearly and/or quadratically. With increasing dietary MHA level, the activities of HSL and CPT-1 decreased linearly. Consistently, the expression levels of lipid synthesis-related genes (including ACC1, ACC2, FAS, and SCD1) were up-regulated in largemouth bass fed the 6.0 and 9.0 g MHA/kg diets, and the transcriptional levels of lipid degradation-related genes (ATGL, HSLa, HSLb, and CPT1) were down-regulated in largemouth bass fed the 6.0 and 9.0 g MHA/kg diets. These results suggested that dietary MHA promoted lipid deposition in the liver by regulating lipid metabolism. A correlation analysis showed that the expressions of ACC1, FAS, SCD1, HSLa, and CPT1 were positively correlated with their corresponding enzyme activities (r_ACC1_ = +0.956, *p* = 0.044; r_FAS_ = +0.976, *p* = 0.024; r_SCD1_ = +0.953, *p* = 0.047; r_HSLa_ = +0.988, *p* = 0.012; r_CPT1_ = +0.0.818, *p* = 0.091, Table 5). These results also indicated that dietary MHA increased the activities of lipogenic enzymes and decreased the activities of lipolytic enzymes by regulating their gene expressions. As far as we know, no information is available regarding the impact of MHA on the activities of lipid-metabolism-related enzymes in the liver. It has been reported that L-carnitine, a metabolite of MHA, reduced the activities of FAS and enhanced the activities of CPT1 in the livers of fish [24]. The reason for the different effects of MHA and its metabolite carnitine on the activities of lipid metabolism enzymes needs further study. Previous studies in cobia, rainbow trout, and tiger puffers found that a high Met supplemental level increased hepatic lipid accumulation. Dietary Met led to an increase in lipolytic gene expression and a decrease in lipogenetic gene expression [4,5,7].

SREBP-1c is the main transcription factor that mediates the expression of lipogenesis genes, such as ACC, FAS, and SCD in liver of fish [7,10]. Conversely, PPARα plays a crucial role in fatty acid oxidation by upregulating genes involved in mitochondrial and peroxisomal β-oxidation, including CPT1 [39]. In this study, with the increasing dietary MHA levels, the mRNA expressions of SREBP-1c increased linearly and the mRNA expressions of PPARα decreased linearly. These results are consistent with the observations in cobia [7] and rainbow trout [14]. Correlation analysis showed that the expression of SREBP-1c was positively correlated with lipogenesis genes (r_ACC1_ = +0.977, *p* = 0.023; r_ACC2_ = +0.981, *p* = 0.019; r_FAS_ = +0.963, *p* = 0.037; r_SCD1_ = +0.932, *p* = 0.068), while the expression of PPARα was positively correlated with CPT1 expression (r_CPT1_ = +0.942, *p* = 0.058, Table 5). These results suggested that dietary MHA increased the expression of lipogenic genes, and decreased that of lipolysis genes partly by regulating the expression of SREBP-1c and PPARα, respectively.

The AMPK is an important integrator of signals that control the energy balance [40]. Previous studies demonstrated that AMPK activation suppressed the activities of SREBP-1c, and promoted PPARα and downstream fatty acids β-oxidation-related gene expression [41,42,43,44]. In the present study, the liver mRNA expressions of AMPKα1 and AMPKα2 linearly and quadratically decreased, respectively. The ratio of P-AMPK/T-AMPK/β-actin linearly decreased with increasing dietary MHA levels. Similar results were also found in rainbow trout [14]. Correlation analysis found that the expression of AMPKα was positively correlated with PPARα (r_PPARα_ = +0.962, *p* = 0.038), and negatively correlated with SREBP-1c (r_SREBP-1c_ = −0.964, *p* = 0.036). These results suggested that dietary MHA up-regulated the mRNA expressions of SREBP-1c and down-regulated the mRNA expressions of PPARα partly via AMPK. In terrestrial animals, the SIRT1/AMPK signaling pathway plays a crucial role in hepatic lipid metabolism. Activation of this pathway exerts inhibitory effects on fatty acid uptake and synthesis while promoting fatty acid β-oxidation [45,46]. In this study, we identified that the mRNA expression of SIRT1 and the ratio of SIRT1/β-actin linearly decreased with increasing dietary MHA levels. A correlation analysis showed that the expression of SIRT1 was positively correlated with AMPKα1 (r_AMPKα1_ = +0.947, *p* = 0.053), suggesting that dietary MHA promotes lipid accumulation in the liver, partly via repressing the SIRT1/AMPK signaling pathway. Grant et al. (2016) reported that a Met restriction up-regulated the mRNA expression of SIRT1 in the kidneys of mice [21]. Chan et al. (2018) also found that Met repressed the mRNA expression and protein level of SIRT1 in mouse aortic endothelium [20]. These results suggest that MHA might have a similar function to Met in regulating the SIRT1 gene expression of fish. However, the detailed mechanism by which MHA regulates lipid synthesis and lipid oxidation via the SIRT1/AMPK pathway in fish requires further investigation.

## 5. Conclusions

In summary, the present study demonstrated that dietary MHA increased hepatic lipid vacuoles and lipid content. Furthermore, supplying dietary MHA could enhance enzyme activity parameters and gene expression related to lipid synthesis, which reduce enzyme activity parameters and gene expression related to lipid oxidation. Dietary MHA-induced lipid accumulation was mediated, at least in part, through the regulation of the SIRT1/AMPK signaling pathway. These findings provide new insights into the impact of MHA on hepatic lipid metabolism in fish, helping to fill the existing knowledge gaps in this area.

## Figures and Tables

**Table 1:** Composition and nutrient content of diets (g/kg).

Ingredients	Dietary MHA Level			
	0.0	3.0	6.0	9.0
Fish meal	250.0	250.0	250.0	250
Soybean meal	220.0	220.0	220.0	220.0
Corn protein meal	100.0	100.0	100.0	100.0
Gelatin	60.0	60.0	60.0	60.0
Soybean oil	100.0	100.0	100.0	100.0
Wheat flour	165.0	162.0	159.0	156.0
CaH_2_PO_4_	40.0	40.0	40.0	40.0
Methionine hydroxy analog	0.0	3.0	6.0	9.0
Vitamin premix ^1^	10.0	10.0	10.0	10.0
Mineral premix ^2^	20.0	20.0	20.0	20.0
Amino acid premix ^3^	11.8	11.8	11.8	11.8
Choline chloride (50%)	10.0	10.0	10.0	10.0
Ethoxyquin (30%)	0.5	0.5	0.5	0.5
Microcrystalline cellulose	12.7	12.7	12.7	12.7
Nutrients content ^4^				
Crude protein	419.6	417.8	417.7	416.7
Crude lipid	124.6	126.8	127.0	125.2
Crude ash	105.8	106.2	105.7	106.4
Moisture	96.2	94.1	93.7	94.9

^1^ Vitamin premix (IU or mg/kg diet): biosterol, 18,000 IU; vitamin D_3_ 9000 IU, 0.001; vitamin K_3_, 16.56; thiamine, 20.03; riboflavin, 54; vitamin B_6_, 33.21; cyanocobalamin, 0.27; α-tocopherol, 28.00; ascorbic acid, 150.00; niacinamide, 26.00; calcium pantothenate, 25.00; folic acid, 1.00; biotin, 0.06; inositol, 400.00. ^2^ Mineral premix (mg/kg diet): Cu, 5.00; Zn, 37.00; Mn, 7.00; Fe, 30.00; I, 1.10; Se, 0.25; Co, 0.20; Mg, 600.00. ^3^ Amino acid premix (g/kg diet): histidine, 0.95; isoleucine, 1.97; lysine, 1.67; valine, 1.69; cystine, 2.08; tyrosine, 2.88; threonine, 2.69. ^4^ Crude protein, crude fat and ash was measured using the Association of Official Analytical Chemists Methods.

**Table 2:** Amino acid composition of experimental diets (on DM-basis, g/kg).

MHA Levels	0.0	3.0	6.0	9.0
Thr	16.6	16.4	17.1	16.8
Val	18.5	18.7	17.9	19.2
Met	8.3	11.0	13.6	16.3
Cys	6.5	6.2	6.7	6.4
Ile	15.5	16.4	16.0	16.7
Leu	31.0	30.5	31.3	31.6
Phe	15.7	16.2	15.8	16.4
Tyr	12.9	12.7	12.1	12.5
His	10.6	10.2	10.8	10.3
Lys	20.1	20.6	20.8	19.9
Arg	22.5	22.7	22.2	22.6
Trp	3.4	3.6	3.3	3.1

Thr: Threonine; Val: Valine; Met: Methionine; Cys: Cysteine; Ile: Isoleucine; Leu: Leucine; Phe: Phenylalanine; Tyr: Tyrosine; His: Histidine; Lys: Lysine; Arg: Arginine; Trp: Tryptophan.

**Table 3:** Primer sequences and optimal annealing temperatures (OAT, °C) of genes selected for analysis by real-time PCR.

Name	Sequences	OAT	Accession Number
ACC1-QF	GGCTCAGTGATGGAGGTCTAT	62.3	MW465382
ACC1-QR	GGTGATCGTAGCAGTGAAGGA		
ACC2-QF	TGGAAAGGGTATCCGTAAAGT	61.8	MW435454
ACC2-QR	AGCAGTCTCGTCCGAACAG		
FAS-QF	CAGGTCTGTACGGTCTTCCA	62.2	MW465391
FAS-QR	CGGGTTCACTCCTCCATCTA		
SCD1-QF	TATCTTTGAATGGGCTCGTGA	60.1	MW465401
SCD1-QR	AACTTTGTCGGCGTACAGGTC		
ATGL-QF	GGAATCTCAGACAACCTGCCTC	62.7	XM_038705351
ATGL-QR	GGTGGAGTGAACTGGATGCTT		
HSLa-QF	TGAACGCATTACCCAGAACC	61.8	MW465409
HSLa-QR	GTGTAGAGTCACAGGAGGCAAA		
HSLb-QF	TCGTCTCCCTGCCTCCTAAT	62.3	MW465407
HSLb-QR	GGCGTAGAAACACTCCTCCAG		
CPT1-QF	TTCCCCTTTATTGAC	60.0	XM_038705335.1
CPT1-QR	AGAACTTCCCTTTGTC		
PPARα-QF	TGGAGCTGGATGACACTGACC	59.0	MK388672.1
PPARα-QR	GAGCCGTAGTGCCTGAACAAT		
SREBP-1c-QF	CCTCCCAGTCCTTTGCTATTG	57.9	MW465403
SREBP-1c-QR	TCCTTGGAGCCAGTTGATGA		
AMPKα1-QF	CCTGAAGGAGGTATGTGACAAG	60.7	MW465410
AMPKα1-QR	CAATGATGAGATGGTAGGCAAC		
AMPKα2-QF	GAGGCGTCTTCTACATCCCA	58.7	MW465405
AMPKα2-QR	GGTCCTGCTTAAACCATTCAT		
LKB1-QF	CTACCAGCCACGGAGGAAG	62.7	MW465396
LKB1-QR	TGCGGCATAGTGTCTCTGAGT		
SIRT1-QF	TACCAGAACAGCCACCAAGT	58.7	MW465402
SIRT1-QR	CATTATTACCAGCAGTCTCCGT		
β-actin-QF	CCCCATCCACCATGAAGA	55.7	AF253319.1
β-actin-QR	CCTGCTTGCTGATCCACAT		
18S-QF	TGAATACCGCAGCTAGGAATAATG	59.0	MH018569.1
18S-QR	CCTCCGACTTTCGTTCTTGATT		

**Table 4:** The activities (U/mg protein) of ACC, FAS, SCD-1, HSL, and CPT-1 in livers of largemouth bass fed diets with graded levels of MHA (g/kg) for 84 days ^1^.

Items ^2^	Dietary MHA Levels, g/kg				*Pr > F* ^2^		
	0.0	3.0	6.0	9.0	ANOVA	Linear	Quadratic
ACC	65.86 ± 4.70 ^a^	77.54 ± 4.73 ^b^	80.88 ± 5.05 ^b^	86.96 ± 2.54 ^c^	0.00	<0.0001	0.06
FAS	48.86 ± 5.44 ^a^	61.36 ± 2.72 ^b^	73.29 ± 3.25 ^c^	75.25 ± 2.25 ^c^	0.00	<0.0001	0.00
SCD-1	52.93 ± 3.03 ^a^	64.24 ± 3.10 ^b^	84.37 ± 9.13 ^c^	89.49 ± 7.11 ^c^	0.00	<0.0001	0.14
HSL	205.04 ± 14.56 ^c^	164.07 ± 6.40 ^b^	155.73 ± 6.52 ^b^	133.59 ± 13.79 ^a^	0.00	<0.0001	0.06
CPT-1	243.67 ± 4.24 ^c^	212.75 ± 4.36 ^b^	179.42 ± 10.19 ^a^	168.79 ± 7.20 ^a^	0.00	<0.0001	0.15

^1^ With nine fish in each group. Values within the same rows having different superscripts are significantly different (*p <* 0.05). ^2^ Acetyl-CoA carboxylase (ACC), fatty acid synthetase (FAS), stearoyl-coenzyme A desaturase-1 (SCD-1), hormone sensitive lipase (HSL), and carnitine palmitoyl transferase-1 (CPT-1).

**Table 5:** Correlation coefficients of some parameters.

Independent Parameters	Dependent Parameters	Correlation Coefficients	*p*
ACC	ACC1 mRNA	0.956	0.044
FAS	FAS mRNA	0.976	0.024
SCD1	SCD1 mRNA	0.953	0.047
HSL	HSLa mRNA	0.988	0.012
CPT-1	CPT-1 mRNA	0.818	0.091
SREBP-1c mRNA	ACC1 mRNA	0.977	0.023
	ACC2 mRNA	0.981	0.019
	FAS mRNA	0.963	0.037
	SCD1 mRNA	0.932	0.068
PPARα mRNA	ATGL mRNA	0.984	0.016
	HSLa mRNA	0.988	0.012
	CPT-1 mRNA	0.942	0.058
AMPKα1 mRNA	SREBP-1c mRNA	−0.964	0.036
	PPARα mRNA	0.962	0.038
	SIRT1 mRNA	0.947	0.053

## Data Availability

The data presented in this study are available in this manuscript.
